# Supplementary material for: First-Year Implementation of the EXercise for Cancer to Enhance Living Well (EXCEL) Study: Building Networks to Support Rural and Remote Community Access to Exercise Oncology Resources
Source: Int J Environ Res Public Health. 2023 Jan 20;20(3):1930. doi: 10.3390/ijerph20031930 (PMC9915392; doi:10.3390/ijerph20031930)
Supplement: Supplementary file 1 [file ijerph-20-01930-s001.zip › ijerph-2104164-supplementary.pdf]

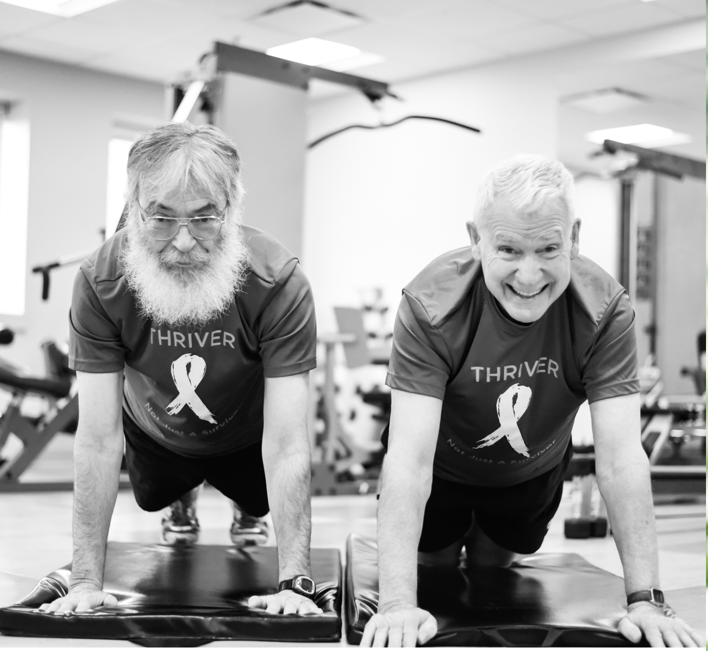

CONTACT INFORMATION

Health & Wellness Lab:

E: wellnesslab@ucalgary.ca

Ph: 403-210-8482

Web:
thriveforcancersurvivors.com/

Please get in touch to learn more.

UNIVERSITY OF CALGARY
FACULTY OF KINESIOLOGY
Health and Wellness Lab

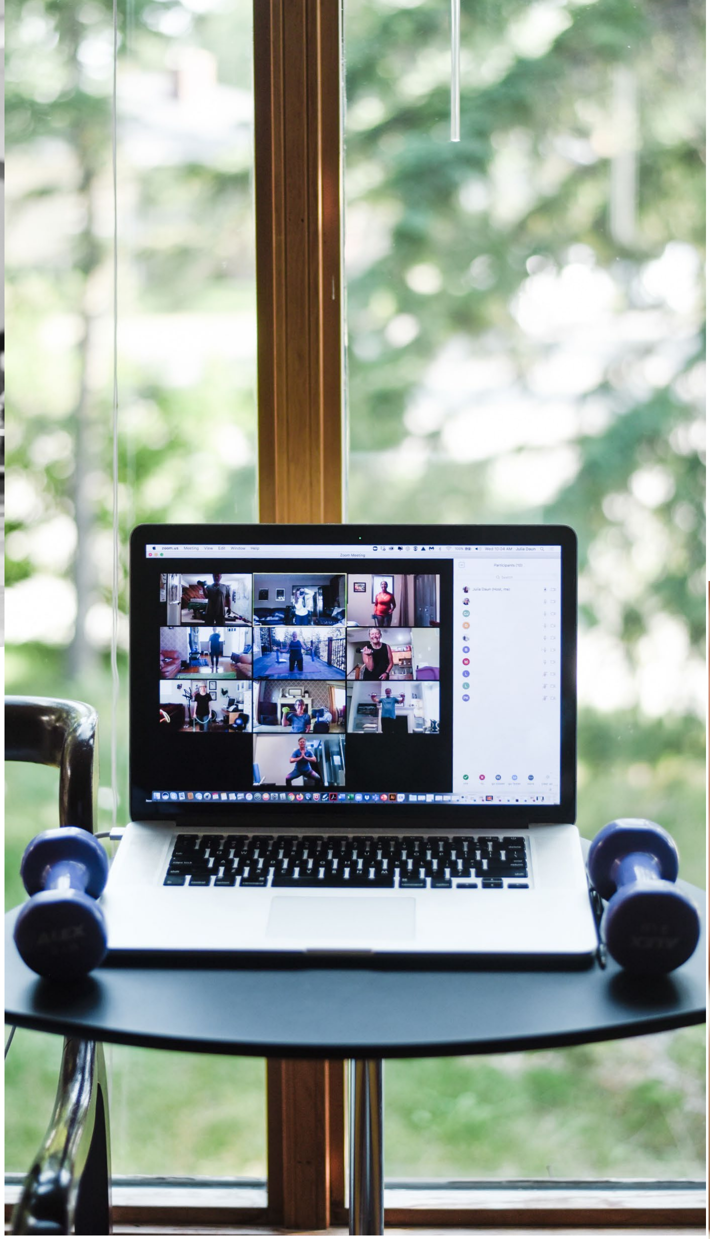

Funding for this study provided by:

Canadian Cancer Society

CIHR IRSC Canadian Institutes of Health Research / Instituts de recherche en santé du Canada

Alberta Cancer FOUNDATION

This study has been approved by the Health Research Ethics Board of Alberta: HREBA.CC-20.0098

Version date: June 9, 2022, V3

File S1

EXCEL

EXercise for **C**ancer to **E**nhance **L**iving Well

EXCEL is the **EX**ercise for **C**ancer to **E**nhance **L**iving Well study. This study provides an exercise program to Canadians living with and beyond cancer.

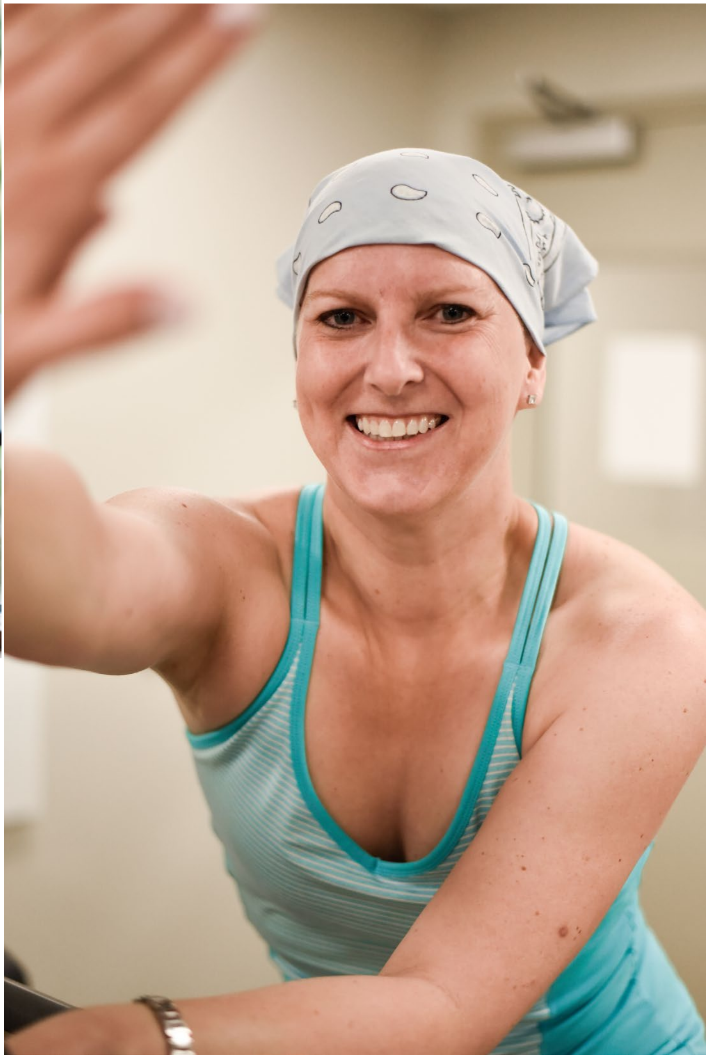

BENEFITS OF EXERCISE:

PHYSICAL:

- Decreases fatigue
- Improves physical function
- Improves ability to do activities of daily living

PSYCHOSOCIAL:

- Boosts energy and mood
- Enhances sleep
- Increases emotional & social well-being

ELIGIBILITY

To be eligible, participants must:

- Have/had a cancer diagnosis
- Be pre-cancer treatment, receiving treatment, or within 3 years treatment completion
- Able to participate in mild/moderate physical activity
- 18+ yrs old and able to consent in English

STUDY ASSESSMENTS

Participants will complete a fitness assessment before and after the exercise program. Each assessment will take approximately 30 minutes, will be assessed either in-person or online through a secure video-conference platform, and will include:

- Medical history and demographic information.
- 6-minute walk test or 2-minute step test to assess aerobic fitness
- Muscular endurance tests
- Core and shoulder flexibility test
- A standing balance test
- Height and weight measurements
- Questionnaires to assess individual symptoms, quality of life, and physical activity levels. Questionnaires will be completed at baseline, after the exercise program, 24-weeks, at 1 year, and annually for up to 5 years.

FAQ

HOW LONG IS THIS STUDY?

Participation in this study includes an 8 to 12-week exercise program (depending on the program offered in your area), two fitness assessments, a series of questionnaires completed four times throughout the year, and follow-up questionnaires annually for up to 5 years.

HOW MUCH DOES IT COST?

There is no cost associated with enrollment in this study; however, participating at some partnering facilities may result in indirect costs such as parking fees.

WHAT ELSE SHOULD I KNOW?

If you would like to know more about the EXCEL study as a potential participant, please email or call the EXCEL team (see contact information).

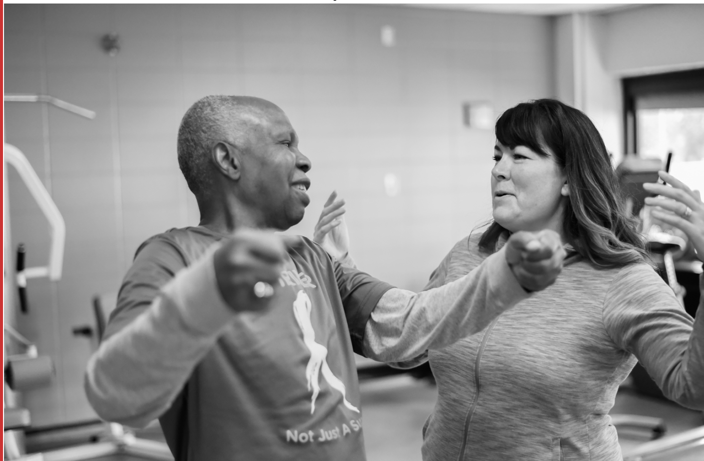

File S2

EXCEL

EXercise for **C**ancer to **E**nhance **L**iving Well

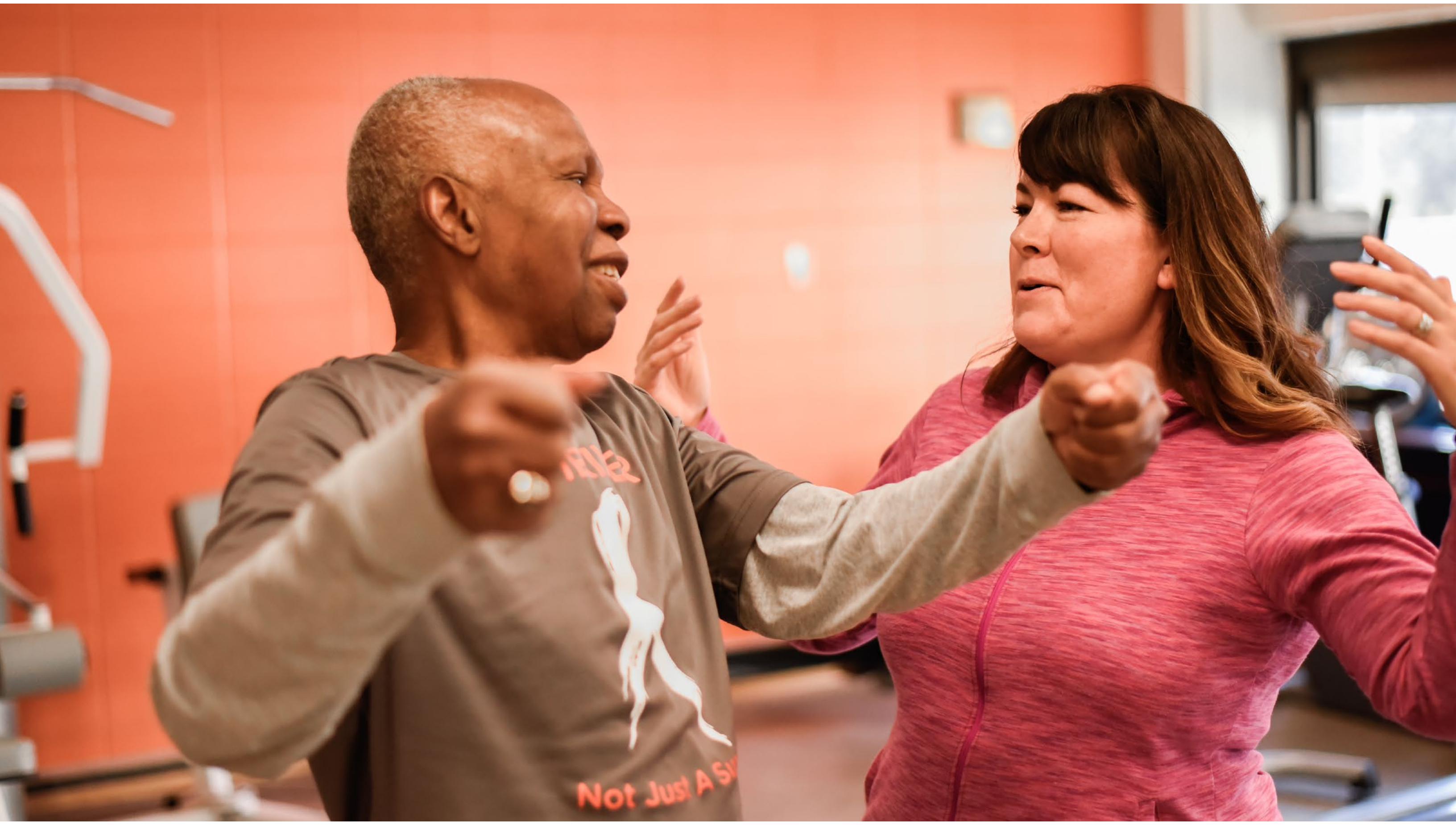

Feeling FATIGUED?

Boost your ENERGY by joining this FREE cancer exercise study

For more information:

Email us at: wellnesslab@ucalgary.ca

Call us at: 403-210-8482

Website: thriveforcancersurvivors.com/

Canadian Cancer Society

CIHR IRSC Canadian Institutes of Health Research / Instituts de recherche en santé du Canada

Alberta Cancer FOUNDATION

UNIVERSITY OF CALGARY FACULTY OF KINESIOLOGY Health and Wellness Lab

Ethics ID: HREBA.CC-20.0098

±ŜǋăƛƻyʃŘl·úŜY WdzyʃŜ ɸúKΣ нлннΣ ±м

File S3
EXERCISE ONCOLOGY REFERRAL

ACE ALBERTA CANCER EXERCISE

EXCEL EXercise for Cancer to Enhance Living Well

UNIVERSITY OF CALGARY
FACULTY OF KINESIOLOGY
Health and Wellness Lab

Physical Activity Screening Form

Patient Label

Last Name: ______________________

First Name: ______________________

Address: ______________________

City: ______________________

Province: ______________________

Postal Code: ______________________

Email: ______________________

Home Phone Number: ______________________

Work Phone Number: ______________________

Cell Phone Number: ______________________

Gender: ______________________

Date of Birth: ______________________

Personal Health Number: ______________________

Current Side Effects:

- ☐ Cardiotoxicity
- ☐ Pulmonary function
- ☐ Fatigue
- ☐ Bowel/bladder changes
- ☐ Neutropenia
- ☐ Anemia
- ☐ Thrombocytopenia
- ☐ Peripheral neuropathy
- ☐ Decreased range of motion
- ☐ Bone or joint issues
- ☐ Lymphedema
- ☐ Skin changes

Other (please indicate below):

Additional Comments:

Patient Information:

Please indicate any significant co-morbidities [that could affect exercise participation]:

Is the cancer metastatic? If so, please provide the locations and stability of metastases.

Physical Activity Clearance:

- ☐ No physical activity at this time
- ☐ Under supervision of qualified exercise professional
- ☐ Unrestricted or progressive physical activity

☐ Modification(s) for exercise (if applicable):

**Physician Signature:**	**Stamp:**	**Date:**

Referral Information: **ACE Program | Fax:** (403) 289-9117 | **Phone**: (403) 210-8482 | **Email:** wellnesslab@ucalgary.ca

File S4

Rural / Remote vs Urban Characteristics

Variable	Rural / Remote (n=199)	Urban (n=37)
Age (years)†	58.1 (12.1)	54.2 (9.9)
Province		
Alberta	113 (56.8)	0 (0.0)
Nova Scotia	58 (29.1)	2 (5.4)
Ontario	28 (14.1)	35 (94.6)
Sex		
Female	158 (79.8)	34 (94.4)
Male	40 (20.2)	2 (5.6)
Ancestral Ethnicity		
Aboriginal	4 (2.0)	0 (0.0)
Asian	7 (3.5)	7 (18.9)
British	70 (35.2)	11 (29.7)
Caribbean	1 (0.5)	0 (0.0)
European	65 (32.7)	7 (18.9)
Latin / Central and South America	4 (2.0)	2 (5.4)
Multi-ethnic	33 (16.6)	5 (13.5)
No information provided	15 (7.5)	5 (13.5)
Education		
High school or less	25 (12.6)	0 (0.0)
More than high school	173 (87.4)	36 (100.0)
Employment Status		
Full Time	32 (16.2)	9 (36.0)
Part Time	26 (13.1)	5 (13.9)
Retired	78 (39.4)	7 (19.4)
Homemaker	8 (4.0)	0 (0.0)
Disability	45 (22.7)	11 (30.6)
Temporarily Unemployed	9 (4.5)	4 (11.1)
Marital Status		
Not Married	44 (22.2)	11 (30.6)
Married	133 (67.2)	19 (52.8)
Common Law	21 (10.6)	6 (16.6)
Cancer Type		
Breast	103 (51.8)	23 (62.2)
Lung	15 (7.5)	5 (13.5)
Hematologic	19 (9.5)	0 (0.0)
Gynecological	11 (5.5)	5 (13.5)
Prostate	15 (7.5)	1 (2.7)
Skin	5 (2.5)	1 (2.7)
Brain	5 (2.5)	1 (2.7)
Colon	6 (3.0)	0 (0.0)
Head and Neck	5 (2.5)	0 (0.0)
Other‡	15 (7.5)	1 (2.7)
Advanced Cancer		
Yes	54 (27.3)	10 (27.8)
No	144 (72.7)	26 (72.2)
Treatment Status		
On	107 (54.0)	21 (58.3)
Off	91 (46.0)	15 (41.7)
Treatment Type for participants ‘On’ treatment		
Chemotherapy	30 (28.0)	3 (14.3)
Radiation	5 (4.7)	0 (0.0)
Hormone Therapy	44 (41.1)	8 (38.1)
Combination*	14 (13.1)	2 (9.5)
Other**	14 (13.1)	8 (38.1)

† *= Age is expressed as mean (standard deviation)*

‡ *Other = bladder, appendiceal, kidney, pancreatic, small bowel, colorectal, gastric, rectal, esophagus*

**Combination = any combination of chemotherapy, radiation, hormone therapy, or "other"*

***Other = immunotherapy, targeted therapy, surgery, antimetabolites*

Rural / Remote vs Urban Implementation Summary

Variable	Rural / Remote	Urban
Reach (n)		
Enrolled Participants	199	37
Direct HCP-Referral	19	1
Indirect HCP Referral	36	1
Self-Referral	144	35
Implementation (%)		
Exercise Intervention Adherence	78.3%	77.8%
Fitness Assessment Completion		
Baseline	98.0%	100.0%
12-week	91.5%	81.1%
Patient-Reported Outcome Completion		
Baseline	97.5%	91.9%
12-week	86.4%	94.6%

Rural / Remote vs Urban Referral Sources

**Referral Resource Descriptions**	**Number of times resources were indicated**	
	**Rural / Remote (n=199)**	**Urban (n=37)**
Word of Mouth		
Friend or Family Member	2	1
Previous or Current Participant	21	0
Support Group	32	2
Health and Wellness Organizations and Programs	49	27
EXCEL Team Outreach		
Online Presentations	1	0
Social Media or Websites	18	4
Study Staff	26	1
Healthcare Provider		
Social Worker	1	0
Lymphedema Specialist	1	0
HCP provided brochure in clinic	3	0
Patient Navigator	3	0
Care Coordinator	3	0
Primary Care Network	5	0
Oncologist	15	1
Nurse	16	0
Print Materials		
Posters	3	0
Brochures	5	0

[*] = participants could indicate multiple referral resources if they chose to answer the question “How did you first find out about the EXCEL program?”

File S5

Supplemental Table 1: HCP Network Organizations (n = 163)

**Alberta (n=55)**	**Nova Scotia (n=21)**	**Ontario (n=87)**
1. Alberta Heartland PCN	1. Cumberland Regional Health Center	1. Anson General
2. Aspen PCN	2. Antigonish Cancer Centre	2. Atikokan General Hospital
3. Barrhead Cancer Centre	3. Bridgewater Hospital	3. Bingham Memorial Hospital
4. Beaumont Family Medical Associates	4. Charlotte County Oncology	4. Blanche River Health
5. Big Country PCN	5. Community Health Team Bedford/Sackville	5. Blue Water Health
6. Bighorn PCN	6. Hants Health & Wellness Team	6. Brampton Civic Hospital
7. Bonnyville Cancer Centre	7. Labrador Health Centre	7. Brantford General Hospital
8. Bonnyville PCN	8. Living Beyond Cancer	8. Cambridge Memorial Hospital
9. Bow Valley Cancer Centre	9. Nova Scotia Health Authority	9. Cancer Centre of Southeastern Ontario
10. Bow Valley PCN	10. St John Regional Oncology	10. Central Regional Cancer Program
11. Bow Valley PCN	11. Colchester Easts Hants Health Center	11. Credit Valley Hospital
12. Calgary Rural Primary Care Network (High River)	12. Cape Breton Cancer Center	12. Department of Women's and Children's Health
13. Calgary Rural Primary Care Network (Strathmore)	13. Westside Medical Center	13. Dryden Regional Health Centre - Rehabilitation Services
14. Camrose Cancer Centre	14. Aberdeen Hospital	14. Durham Regional Cancer Centre
15. Camrose PCN	15. Valley Regional Hospital	15. Emily Anderson Memorial Menoyawin Health Centre
16. Care Plus Medical Clinic	16. Colchester Community Hospital	16. Espanola Regional Health Center
17. Chinook PCN	17. East Hants Medical Center	17. Gateway Centre of Excellence in Rural Health
18. Cold Lake PCN	18. South Shore Regional Hospital	18. Grand River Regional Cancer Centre
19. Drayton Valley Cancer Centre	19. Nova Scotia Cancer Center	19. Grey Bruce Health Services
20. Drayton Valley PCN	20. Cancer Care Nova Scotia	20. Headwaters Specialist Clinic
21. Drumheller Cancer Centre	21. Wolfville Professional Center	21. Health Sciences North Cancer Centre
22. Edmonton Southside PCN		22. Health Sciences North Regional Cancer Program
23. Grande Prairie Cancer Centre		23. Home and Community Care Support Services
24. High River Cancer Centre		24. Hopital Notre-Dame Hospital Physio
25. Highland PCN		25. Javinski Cancer Centre
26. Hinton Cancer Centre		26. Joseph Brant Hospital Cancer Clinic

27. Jack Ady Cancer Centre
28. Kalyna PCN
29. Lakeland PCN
30. Lamont Office
31. Leduc Beaumont Devon PCN
32. Lloyd PCN
33. Lloydminster Community Cancer Centre
34. Margery E. Yuill Cancer Centre
35. McLeod River PCN
36. Northwest PCN
37. Palliser PCN
38. Prostate Cancer Centre
39. Peace River Cancer Centre
40. Peace River PCN
41. Peaks to Prairie PCN
42. Peter Lougheed Centre
43. Provst PCN
44. Red Deer PCN
45. Redwater Office
46. Rocky Mountain House PCN
47. Saddle Hills PCN
48. Sherwood Park Strathcona Country PCN
49. St Alberta and Sturgeon PCN
50. Tom Baker Cancer Centre
51. Wainwright PCN
52. WestView PCN
53. Wetaskiwin PCN
54. Wolf Creek PCN
55. Wood Buffalo PCN

27. Juravinski Cancer Centre
28. Kasabonika Lake First Nation
29. Kenora Physiotherapy & Sports Injury Clinic
30. Kingston General Hospital
31. Kingston Health Sciences Centre
32. Kitchenuhmaykoosib Inninuwug - Nursing Station
33. Lady Dunn Health Centre
34. Lake of the Woods District Hospital
35. Lakeridge Health
36. Lakeridge Health Corporation
37. Lakeridge Health Oshawa
38. LHSC London Regional Cancer Program
39. London Health Sciences Centre
40. London Regional Cancer Centre
41. Lyrette Physiotherapy
42. Mackenzie Health
43. Mackenzie Richmond Hill Hospital
44. Manitoulin Health Centre
45. Markham Stouffville Hospital
46. Mary Berglund Community Health Centre
47. Mattawa Hospital Physio
48. MICS Groups of Health Services
49. Mississauga First Nation – Red Pine Lodge
50. Nellie Fiddler Memorial Health Centre
51. Northeast Cancer Centre
52. Ottawa General Hospital
53. Ottawa Hospital Cancer Centre
54. Peel Regional Cancer Centre
55. Queensway Health Center Site

56. Resiliance Physiotherapy
57. Rouge Valley Centerary Hospital
58. Royal Victoria Hospital
59. Royal Victoria Regional Health Centre
60. RSM Durham Regional Cancer Centre
61. Sault Area Hospital
62. Saulte Ste Marie Academic Medical Association
63. Scarborough Grace Hospital
64. Scarborough Health Network
65. Sensenbrenner Hospital
66. Simcoe Muskoka Regional Cancer Program
67. Sioux Lookout Meno Ya Min Health Centre
68. Southlake Regional Health Centre
69. St. Joseph's General Hospital
70. St. Joseph's Health Centre
71. Stronach Regional Cancer Centre
72. Sunnybrook Odette Cancer Centre
73. The Credit Valley Hospital
74. The Ottawa Hospital
75. The Ottawa Hospital Cancer Centre
76. Thunder Bay Regional Health Centre
77. Timmins and District Hospital
78. Trillium Health Partners
79. Trillium Health Partners - Credit Valley Site
80. Trillium Health Partners Oncology
81. University Health Network
82. Walker Family Cancer Centre
83. Weeneebayko General Hospital Physio
84. William Osler Health System

85. Wilson Memorial General Hospital
86. Windsor Regional Cancer Centre
87. Wright Physiotherapy
